# Fluorescent polymer as a biosensing tool for the diagnosis of microbial pathogens

**DOI:** 10.1038/s41598-024-51919-6

**Published:** 2024-01-25

**Authors:** Selvi Krishnan, Stephen Jose, Bhuvana K. Periyasamy, S. Angayarkanny, R. Joseph Bensingh

**Affiliations:** 1 Central Institute of Petrochemicals Engineering and Technology, Chennai, India; 2 Department of Chemistry, Anna University, Chennai, India

**Keywords:** Biological techniques, Microbiology, Medical research, Chemistry

## Abstract

Diseases and diagnoses are predominant in the human population. Early diagnosis of etiological agents plays a vital role in the treatment of bacterial infections. Existing standard diagnostic platforms are laborious, time-consuming, and require trained personnel and cost-effective procedure, though they are producing promising results. These shortcomings have led to a thirst for rapid diagnostic procedures. Fluorescence-based diagnosis is one of the efficient rapid diagnostic methods that rely on specific and sensitive bacterial detection. Emerging bio-sensing studies on conducting polymers (CPs) are gaining popularity in medical diagnostics due to their promising properties of high fluorescence efficiency, good light stability, and low cytotoxicity. Poly[2-methoxy-5-(2′-ethylhexyloxy)-1,4-phenylenevinylene] (MEH-PPV), is the first identified soluble polymer and model material for understanding the fundamental photophysics of conventional CPs. In this present study, MEH-PPV is used as a fluorescent dye for direct pathogen detection applications by interacting with the microbial cell surface. An optimized concentration of MEH-PPV solution used to confirm the presence of selective bacterial structures. The present study endeavours towards bacterial detection based on the emission from bacteria due to interfacial interaction between polymer and bacterial surface.

## Introduction

The increase in pathogen infections in clinical settings has become a public health problem that has attracted worldwide attention. Fungi and bacteria are the main cause of pathogenic infections, which have very serious consequences, including high medical costs, fatal diseases, and increased patient mortality. Bacteria include Gram-negative and Gram-positive bacteria that have different components on the cell membrane. The pathogenic capacity of bacteria is often related to the components of the cell envelope^1,2^. Fungal infections caused by *Candida albicans* lead to morbidity and mortality in immunocompromised patients^3,4^. In clinical settings, patients suffer from multiple microbial infections caused by multiple pathogens^5^. Urinary tract infections (UTIs) are the second most common cause of recurrent infections affecting patients of all populations^6^. Even with this high incidence, diagnosis is based solely on symptoms and urine culture. Urine dipstick analysis is the traditional technique used to detect nitrite and leukocyte esterase. However, its lack of sensitivity and specificity limits its use in real-time analysis. Moreover, clinical diagnosis requires urine culture and Gram staining, which is laborious and time-consuming. In this context, clinical molecular platforms such as matrix-assisted laser desorption ionization (MALDI-TOF), fluorescence in situ hybridization (FISH), and polymerase chain reaction (PCR) have been developed to overcome major drawbacks of the conventional methods in clinical technology^7–9^. However, these techniques are still time-consuming due to sample processing and isolation of bacteria from urine prior to analysis^10^. Development of targeted and rapid screening for early detection of urinary tract infections is essential. UTIs are mainly caused by *Escherichia coli*, *Klebsiella pneumoniae*, *Proteus mirabilis*, *Enterobacter* species and *Staphylococcus aureus* and are the most common urinary tract pathogens, accounting for 85–90% of cystitis and pyelonephritis cases. *Candida species* are another most common cause of fungal urinary tract infections or candiduria^11,12^. Candida urinary tract infection can originate from the lower urinary tract, in some cases ascend to the kidneys^13^. It is clear that multimicrobial interactions lead to increased severity of urinary tract infections^14^.

Conjugated polymers have been widely used in chemical and biological detection due to their important effects on light harvesting and optical signal amplification^15–17^, fluorescence brightness, fast diffusion rate and low toxicity^18–21^. A conjugated polymer-based detection system for pathogenic microorganisms is an emerging platform in clinical diagnostics. Fluorescence-based pathogen detection has attracted much attention in recent years and provides powerful tools for various applications such as bioimaging, biosensing, and drug delivery^22–26^. Common fluorescent materials include organic dyes, fluorescent nanoparticles, quantum dots (QDs)^27–29^, dye-doped silica colloids, and coloured latex spheres^30^. Recently, light-emitting polymer derivatives with π-conjugated structures have become a rising star among other polymer derivatives due to their attractive performances such as high fluorescence brightness and excellent light stability. MEH-PPV, poly[2-methoxy-5-(2′-ethyl-hexyloxy)-1,4-phenylene-vinylene], an important fluorescent conjugated polymer, is widely used in organic light-emitting diodes (OLEDs), solar cells, biological sensors and other optoelectronic devices are desirable and processable due to its electronic and optical properties^31–35^. In this study, we developed a simple and effective direct pathogen detection method using the fluorescent properties of synthesized MEH-PPV. Further the sensitivity of optimized preparation was evaluated with artificial urine medium (AUM) spiked with known concentrations of microbial cultures. Also, the changes in photophysical spectrum evaluate its interaction with UTI pathogens, and assess its sensitivity and specificity for microbial detection.

## Experimental

### Materials

2-Ethylhexyl bromide was obtained from M/s. Sigma Aldrich Materials, 4-methoxyphenol (98%), acetic acid (99%) and potassium tert-butoxide (98%) were purchased from M/s. Avra, sodium hydride (Paraffin oil 60%) and hexane were provided by M/s. Lobal Chemie, ethyl acetate was received from M/s. Emplura, magnesium sulphate and para-formaldehyde were obtained from M/s. Fisher Scientific, hydrobromic acid (30–33% in acetic acid) was supplied by M/s. Spectrochem, Sodium bicarbonate was purchased from M/s. SDFCL, benzyl bromide (stabilized with propylene oxide) was provided by M/s. TCI, tetrahydrofuran was received from M/s. Rankem, methanol was supplied from M/s. SRL, whatman filter paper 40 ashless was obtained from M/s. GE Healthcare, N,N dimethylformamide and chloroform were purchased from M/s. Qualigens. *Escherichia coli* (ATCC 25922), *Candida albicans* (ATCC 10231), *Staphylococcus aureus* (ATCC 25923), and culture media were purchased from M/s. Himedia laboratories.

### Methods

#### Synthesis of poly[2-methoxy-5-(2-ethylhexyloxy)-1,4-phenylenevinylene] (MEH-PPV)

MEH-PPV can be synthesized through various methods; however the most preferred method is Glich route process, since the process yields polymer with good stability, high molecular weight and low defect as reported by earlier research^36^. The process involves 3 steps as discussed below.

##### Step 1: Preparation of 1-((2-ethylhexyl) oxy)-4-methoxybenzene (MEHB)—(Monomer 1)

0.08 mol of 4-methoxyphenol and 0.9 mol of 2-ethylhexyl bromide was taken in a single neck round bottom flask with the slow addition of dimethyl formamide (DMF) as solvent at room temperature and stirred continuously. 0.16 mol of sodium hydride (NaH) was added to this mixture in batches at 0 °C. The mixture was stirred until it reaches room temperature. The solubility of NaH reduces at higher temperature; hence the temperature of 0 °C is maintained during addition of NaH. After the addition of NaH, nitrogen gas was introduced. After complete dissolution of NaH, slowly the temperature raised to 70 °C and the mixture was refluxed at the temperature of 70 °C and stirred for 12 h continuously by maintaining the nitrogen atmosphere. After 12 h of reaction, the mixture was brought to room temperature. Then the mixture was transferred to a separation funnel and the organic matter was extracted by the addition of ethyl acetate and water. Subsequently, magnesium sulphate was added to eliminate moisture in the mixture. Further it was purified by distillation and finally, a thick brown coloured solution of MEHB was obtained, which was labeled as monomer-I.

##### Step2: Preparation of 1,4-bis(bromomethyl)-2-((2-ethylhexl) oxy)-5-methoxybenzene (MEHDBMB)—Monomer II

0.06 mol of MEHB and 0.001 mol of para-formaldehyde made as solution—1 was taken in a single neck round bottom flask and stirred. A mixture of 0.182 mol of hydrobromic acid (30% HBr in acetic acid) with 0.5 mol of acetic acid made as solution—2 was added drop wise to the round bottom flask and stirred continuously. Then reflux the reaction mixture at 80 °C in the nitrogen atmosphere for 48 h. At the end of the reaction, the mixture was cooled to room temperature. The produced solid mass was diluted with chloroform and later washed with cold water at least twice. After removal of chloroform, the organic layer was separated and dried over magnesium sulphate followed by extraction under reduced pressure. Later it was purified with hexane for recrystallization of the 1,4-bis(bromomethyl)-2-((2-ethylhexyl) oxy)-5-methoxybenzene and pale white colour solid was obtained**.**

##### Step 3: Preparation of MEH-PPV through polymerization of MEHDBMB

In a round bottom flask, 0.001 mol of MEHDBMB was mixed with THF solution with 0.035 mol of potassium tert-butoxide (BuOK) as catalyst. To this mixture, 0.001 mol of benzyl bromide (chain stopper) was added in order to prevent gelation. The mixture was refluxed at the temperature of 70 °C for about 3 h in the nitrogen atmosphere. The product was precipitated by adding 50 mL of methanol and the residue was filtered using Whatman filter paper. The filtered precipitate was washed repeatedly with distilled water until the water is neutralized (pH = 7). The Polymerization reaction yields a red orange colour solid powder, which was died in hot air oven.

#### Characterization of MEH-PPV

Fourier transform infrared (FTIR) spectra were recorded using a Nicolet iS5-6700 model with wavenumber ranging from 4000 to 500 cm^−1^. The UV-visible absorption spectrum was obtained using a Hitachi model U-2900 spectrophotometer. A JY Fluorolog-3-11 model spectrofluorometer was used to measure the photoluminescence, with a range of 180–850 nm, and a resolution of 0.2 nm as the source of light (maximum at specific wavelengths). MLX- Plus Magus laboratory binocular was employed to capture the stained bacteria, that were then examined using a microscope.

#### Microbial culture

The liquid medium was prepared by dissolving 10.0 g of peptone and 5.0 g of NaCl in 1000 mL of distilled water, and the solid medium was prepared by dissolving 17.5 g of peptone, 2 g of meat extract, 1.5 g of starch and 17 g. Agar was prepared in 1000 mL of distilled water, dissolution medium according to the description, pH 7.3–7.5 and autoclaved. Sterile medium used to prepare bacterial suspension. Bovine serum albumin (BSA) was purchased from Media Laboratories. All reagents were of analytical grade and solutions were prepared in double distilled water.

*E. coli*, *C. albicans,* and *S. aureus* were cultured in 50 mL liquid medium on an orbital shaker at a speed of 120 rpm and a temperature of 37 °C for 24 h. A 10 mL bacterial harvest was obtained by washing the cell pellet twice with water by centrifugation at 4000 rpm. Then the harvested bacterial suspensions were stored in a refrigerator at 4 °C for further use. The acquisition method for *C. albicans* is similar to liquid media supplemented with a few drops of BSA to support optimal growth. The stored suspensions were diluted with water/liquid medium to reach the required concentrations for analytical purposes. Colonies obtained directly from culture plates were also used in the experiment. The concentration of bacteria and yeast was determined by the traditional plate counting method.

#### Microscopic preparation

MEH-PPV solution with concentration range of 3 mg mL^−1^ considered as stock. 50–100 µL of MEH-PPV solution was directly pipetted onto a clean microscopic glass slide, followed by the addition of single isolated colony of test organism (*E. coli*/*S. aureus*/*C. albicans*) from 12 to 16 h culture plate. Uniform smear has made with the help of inoculation loop. The smear again flooded with 50–100 µL of MEH-PPV solution to form improved contact between the polymer and the pathogen. Immediately the glass coverslip placed over the slide and observed with the help of light microscope. The maximum time required for sample preparation and microscopic observation is about 3–5 min. MEH-PPV, being light emitting polymer, the emission property elucidated when exposed under UV illumination. Hence, the specimen stage of microscope exposed with handheld UV lamp.

#### Absorption and emission measurements

UV-visible absorption and photoluminesence spectrum was carried by dissolving 3 mg of synthesized MEH-PPV in 3 mL toluene. This is referred to as stock concentration. To obtain the limited spectrum, the stock concentration was further diluted to 1:2 or 1:5 ratios with toluene. The diluted solution was used to record the absorption and emission spectrum. The UV-vis region of energy in the electromagnetic spectrum ranges from 1.5 to 6.2 eV, corresponding to a wavelength range of 700 to 200 nm and the spectrum was obtained by transferring the diluted MEH-PPV solution to the quartz cuvette. The specific excitation wavelength provided for PL spectral studies in order to receive the emission wavelength from the target material. In bacterial evaluation studies, the optimized diluted ratios were used to get an accurate spectral wavelength of bacterial cells. The MEH-PPV and bacterial cell suspension mixed vigorously for a few seconds. The resulting suspension contained maximum of free-floating bacterial cells to record the appropriate photo emission. Both the UV-vis and PL spectra were collected at room temperature.

## Result and discussion

### Characterization of synthesized MEH-PPV

**Figure 1:**
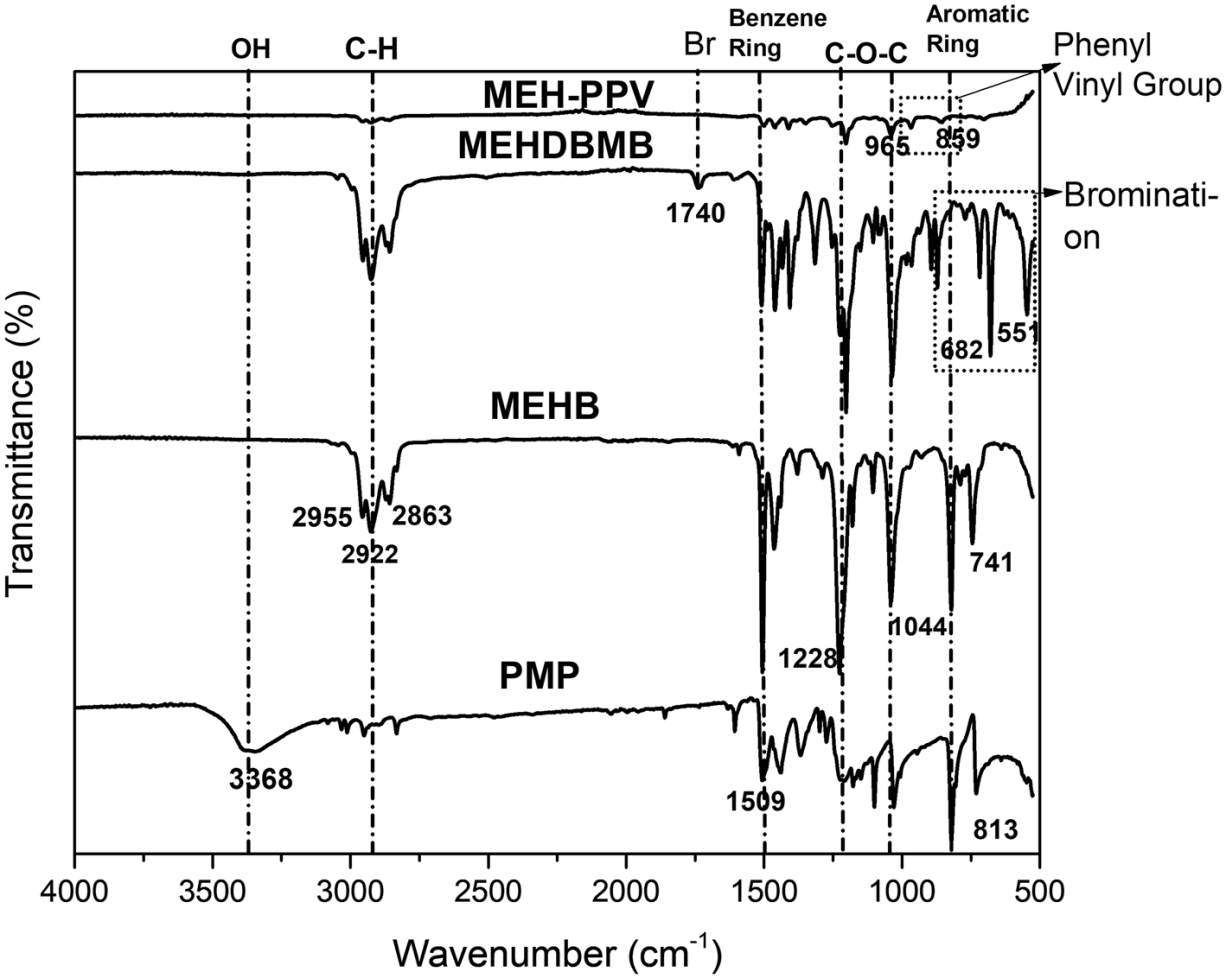
FTIR Spectrum of PMP, MEHB, MEHDBMB, and MEH-PPV.

Figure 1 shows the FTIR spectrum of PMP, MEHB (monomer 1), MEHDBMB (monomer 2) and MEH-PPV (polymer). The peak at 3368 cm^−1^ in PMP (Fig. 1) is due to the presence of OH group on the phenyl ring of p-methoxy phenol, which is observed to be disappeared when 2-ethylhexyloxy side chain was added. The appearance of new bands at 2955 cm^−1^ and 2863 cm^−1^ in MEHB (Fig. 1) denotes the formation of methylene group from 2-ethylhexyl side chain.

The vibration peak at 1509 cm^−1^ signifies the benzene ring. The peaks at 1228 cm^−1^ and 1044 cm^−1^ are due to C–O–C of ester moiety, confirming the replacement of hydroxyl group by an alkoxy group. The peaks at 682 cm^−1^ and 551 cm^−1^ is due to stretching vibrations of Br–C, confirms the reaction of bromomethylation in MEHDBMB. The peak at 551 cm^−1^ was disappeared during polymerization and new peak occurs at 1607 cm^−1^, attributed to long conjugated double bonds. This reveals that the polymerization reaction has taken place. The main characteristic absorption peak at 859 cm^−1^ indicates the out-of-plane phenyl CH wag and the small peak at 965 cm^−1^ denotes the trans double bond vinylene C–H wagging which signifies the dipole normal to the phenyl vinyl plane.

### Photophysical characteristics of MEH-PPV

**Figure 2:**
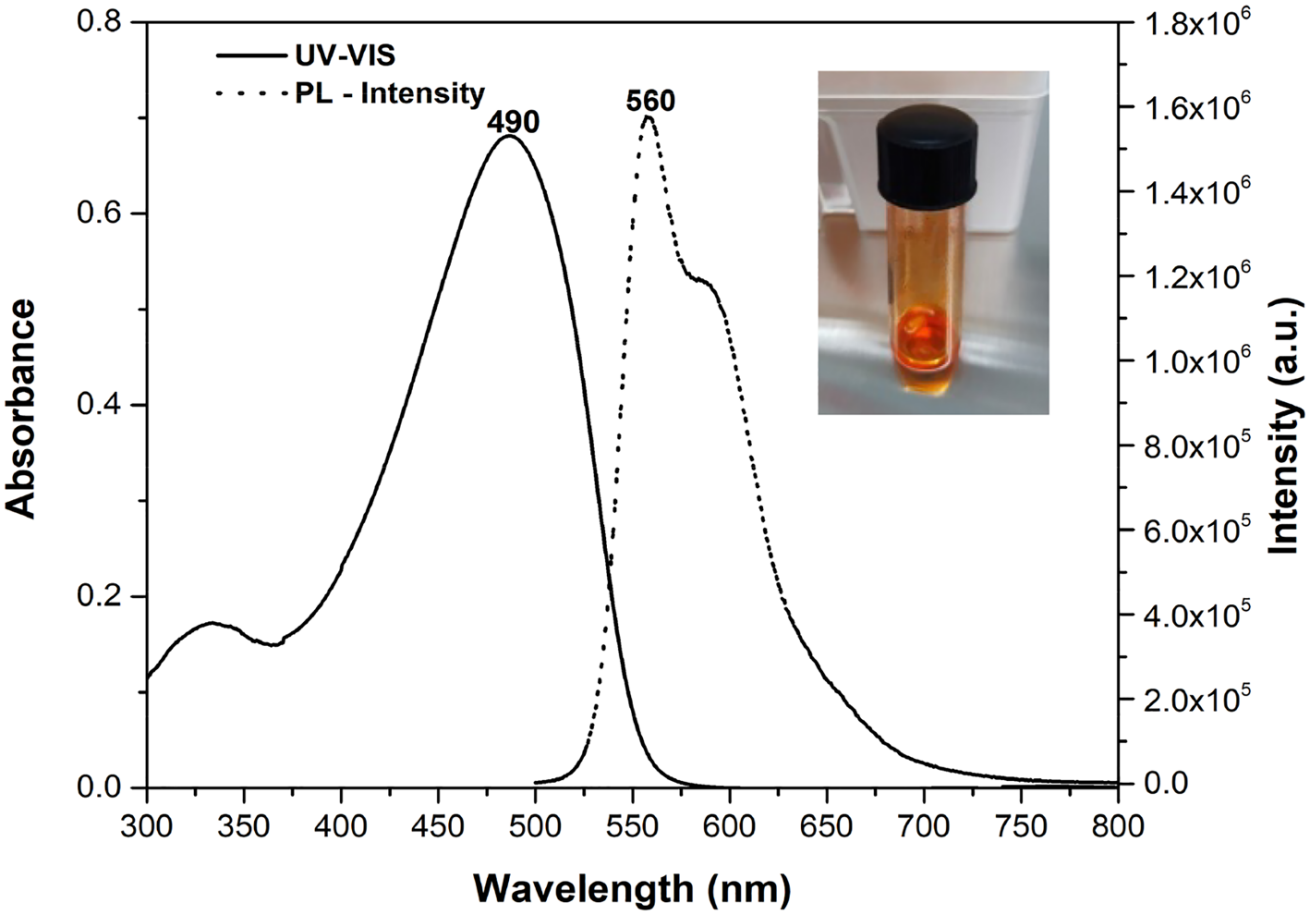
UV–vis and PL spectrum of MEH-PPV in toluene.

Figure 2 shows the UV-visible and Photoluminescence spectrum of MEH-PPV solution. The solution was prepared by dissolving 3 mg in 1 mL of toluene. The UV-vis absorption spectrum of MEH-PPV (solid line) shows a significant absorption at 490 nm with a small shoulder at 355 nm. The absorption is attributed due to transition between the electronic levels of the bonding (π) and antibonding orbitals (π*). The π–π* transition of the conjugated polymer causes the broad absorption band at 490 nm in the UV-vis spectra of MEH-PPV and the short chromophores of MEH-PPV at 335 nm^37^.

The photoluminescence spectra of MEH-PPV solution is depicted in Fig. 2 (dashed line). The spectrum reveals the near band edge emission (NBE) at 560 nm and a small exciton at around 590 nm, which is the characteristic red–orange emission of MEH-PPV^38^. The emission at 560 nm arises due to the relaxation of excited p-electrons to the ground state (intrachain excitation), while the peak at 590 nm is related to the interchange states (interchain interactive eximers).

### Pathogen sensing characteristics of MEH-PPV

#### Selection of solvent

**Figure 3:**
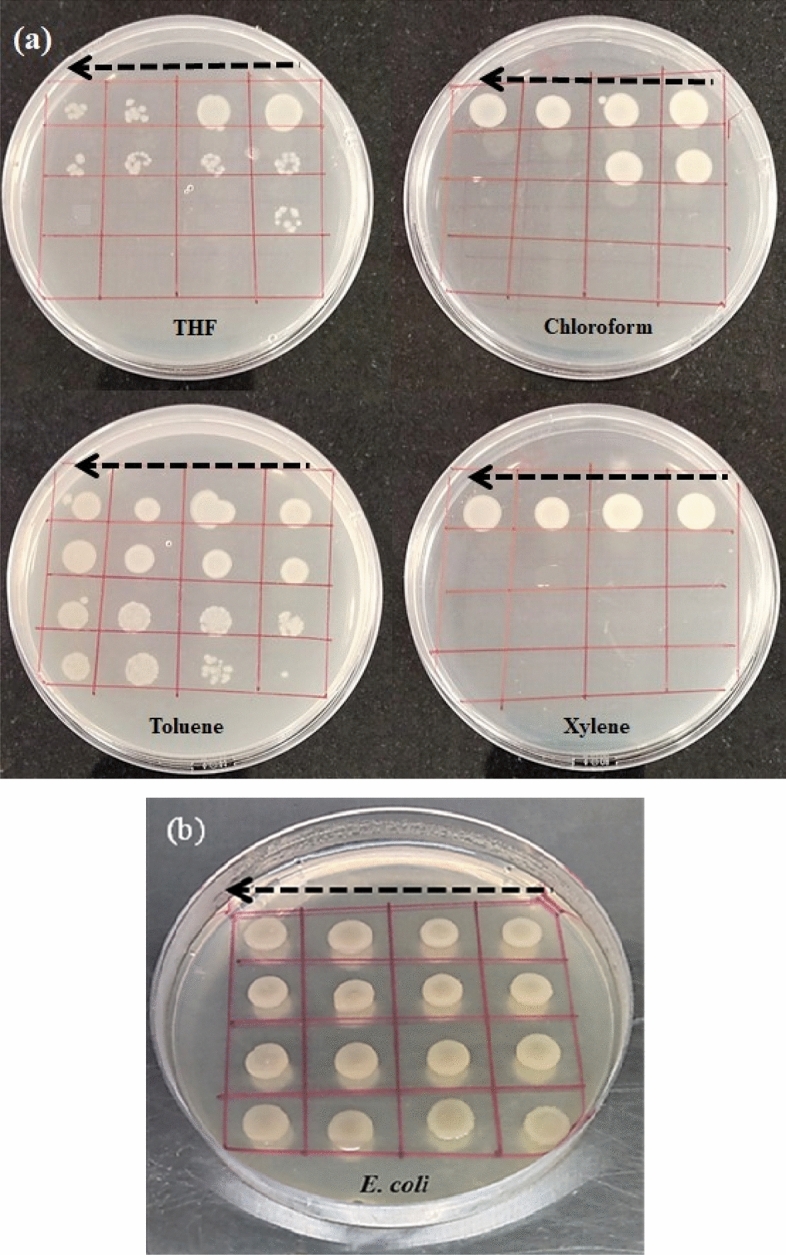
(**a**) Shows the growth of *E. coli* after incubating with solutions of MEH-PPV dissolved in THF, chloroform, toluene, and xylene respectively. The arrow on the digital image shows the direction of inoculum added on the culture plate. (**b**) Shows the control (without MEH-PPV solution) growth with respect to the same time interval of inoculation of test plate.

Selection of appropriate solvent and concentration takes major part in photoluminescence properties of MEH-PPV. MEH-PPV solution with 1 mg mL^−1^, 3 mg mL^−1^, and 5 mg mL^−1^ concentration was prepared using organic solvents such as toluene, xylene, tetrahydrofuran (THF), and chloroform. In order to assess the compatibility of pathogens with MEH-PPV in different solvents, the viability studies were carried out. Bacterial suspension mixed with MEH-PPV solution (in various solvents) was placed on a microscopic slide and viewed through microscope at regular intervals of time, starts from 0 min, 5 min, 15 min, 30 min, 45 min, 1 h and up to 2 ½ h. The study reveals that MEH-PPV in toluene was found considerably more suitable since, organisms are live and stable for upto 2 ½ h, after which slow inactivation of cultured organisms are observed, whereas in other solvents the organisms are found inactive in a shorter time of about less than are equal to 1 ¾ h. To support the microscopic observation of the viability of bacterial cultures, 20 µL from the mixture of MEH-PPV-toluene and bacterial suspension was inoculated at the same interval of time. Inoculated plates were incubated at 37 °C for 24 h. After incubation the plates were macroscopically observed for the presence/absence of bacterial growth (Fig. 3a). The 4 h old bacterial suspension without the addition of MEH-PPV solution is used as growth control (without MEH-PPV) to show the actual growth of bacteria when incubated at the same interval of time (Fig. 3b).

Macroscopic examination of culture plates exhibits the actual inoculum size and the reduction over a period of incubation. There by toluene was found to be least effective against bacterial cells.

#### Optimization of MEH-PPV concentration and pathogen detection efficiency

**Figure 4:**
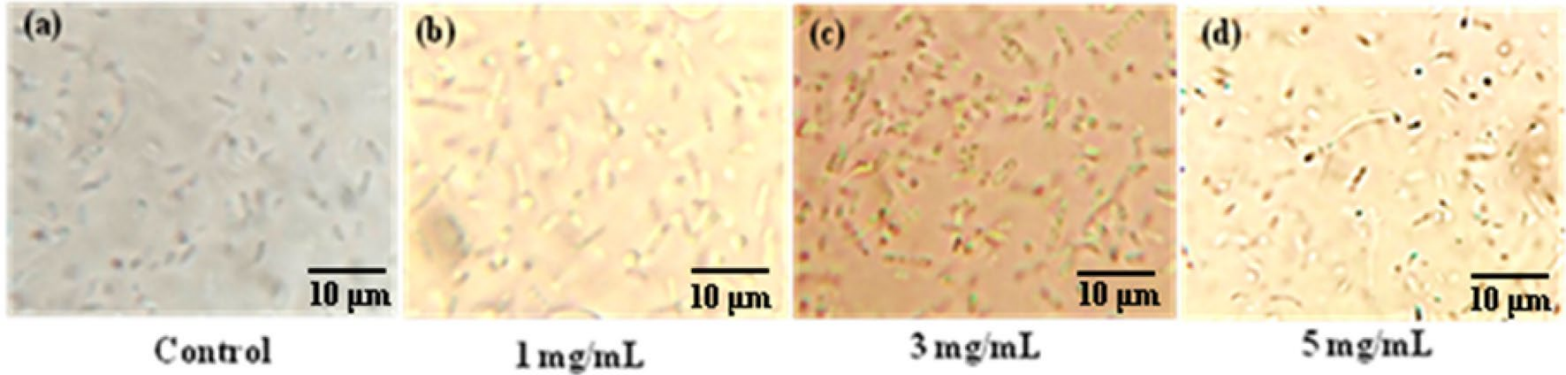
Microscopic image (**a**–**d**) shows the control and luminescence from *E. coli* exposed to different concentration of MEH-PPV solution respectively.

**Figure 5:**
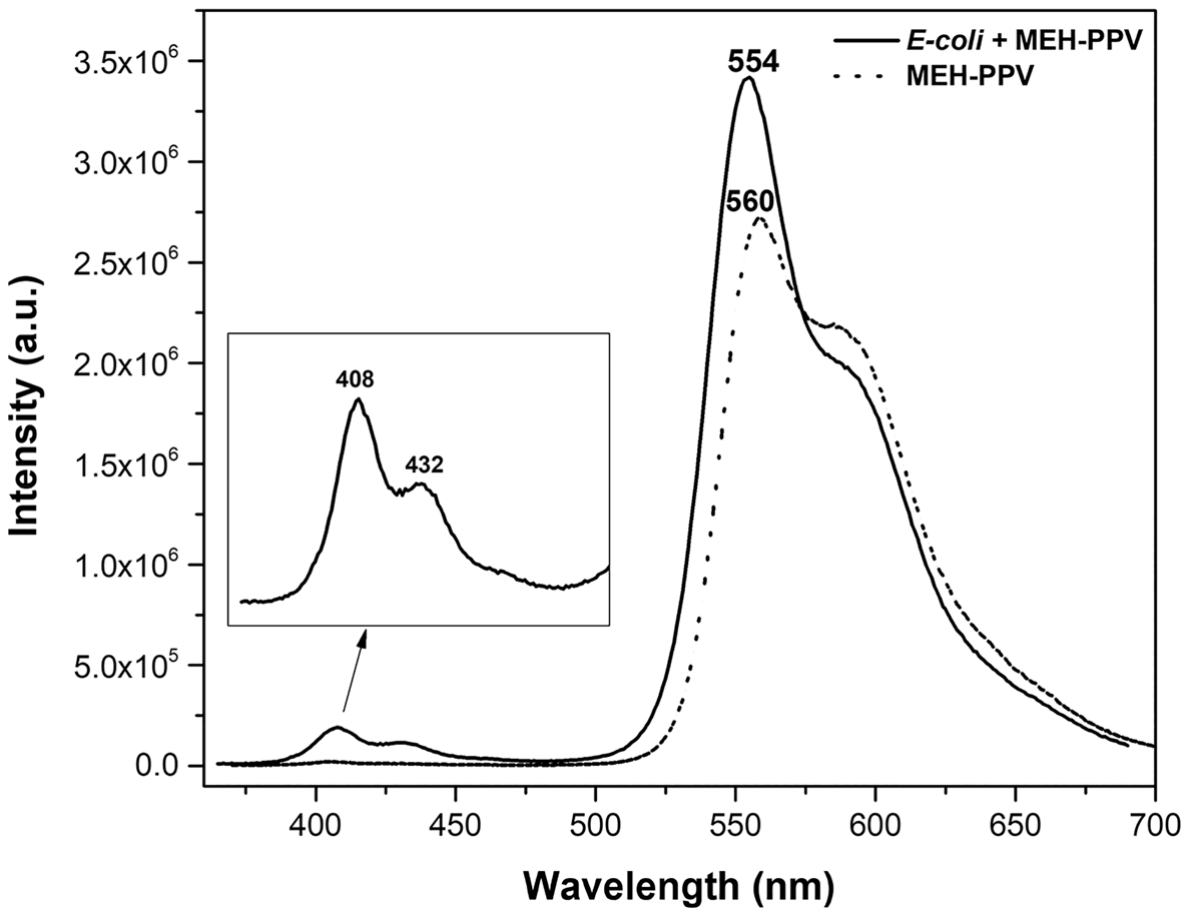
PL spectra of MEH-PPV with (solid line) and without (dashed line) *E. coli.*

From the above study, toluene is found suitable for further analysis. 3 different concentrations of the MEH-PPV solution (1 mg mL^−1^, 3 mg mL^−1^, and 5 mg mL^−1^) were prepared and mixed with *E. coli* suspension having microbial count of 1 × 10^5^ CFU/mL. The suspension was placed on the microscopic glass slide. The same was viewed through microscope under the magnification of 100× and simultaneous illumination of UV-C lamp. Due to the interaction of cell wall of bacteria with MEH-PPV, a green emission was observed from *E. coli* when observed through microscope under the illumination of UV-C radiation. Figure 4 shows the microscopic image observed at 100× magnification. MEH-PPV solution of 1 mg/mL (Fig. 4b) does not shows distinguished emission from bacteria. However, the solution with 3 mg/mL (Fig. 4c) and 5 mg/mL (Fig. 4d) evident the green emission form *E. coli.* Further, as compared to 3 mg/mL, the solution 5 mg/mL does not show prominent emission (Fig. 4d) and, only few *E. coli* has shown the luminescent stains due to poor interaction of *E. coli.* Figure 4a distinguishes between unstained bacterial cells from stained. This is since the MEH-PPV solution does not have adequate interaction with *E. coli*, which occurs due to immiscible nature of aqueous *E. coli* suspension in MEH-PPV-Toluene solution. 3 mg/mL concentration of MEH-PPV solution is found to be optimal for further studies. The pH of the finalized solution concentration is measured about 5.5. Further the optimized concentration of MEH-PPV solution is validated with the highest and lowest concentrations of *E. coli* suspension to observe the sensitivity of prepared dye (Fig. S2) and the obtained results shows the sensitive detection even at low concentration of microbes.

#### Photoluminescence studies for bacterial detection

The photoluminescence spectrum of MEH-PPV and *E. coli* suspension in MEH-PPV solution is depicted in Fig. 5. The MEH-PPV (dashed line) has an emission peak at 560 nm and a shoulder peak at 590 nm, and the peak position more green shifted with MEH-PPV and *E. coli* mixture (solid line). In the MEH-PPV with *E. coli* suspension, the emission peak of *E. coli* recorded at the wavelengths of 408 nm and 432 nm respectively. Among the fluorescence peak at 432 nm considered as sensitive which confirms the binding of MEH-PPV to the bacterial cell surface, while the peak at 408 nm was obtained due to Rayleigh scattering or it would have been resulted due to unbounded or partially bounded *E. coli* cells with MEH-PPV solution at a particular time interval. The hydrophobic nature of the MEH-PPV may remain as a cause when it encounters the bacteria in water. These representing peaks were absent in the PL spectrum of MEH-PPV. To correlate the electron interaction of absorption energy and photon emission in the atoms both PL measurements taken under the same 360 nm excitation. The intensity value of the peak of 560 nm and 554 nm measured as 2.7 × 10^6^ a.u and 3.4 × 10^6^ a.u respectively. This clear increase in intensity of the mixed solution due to release of excessive photon from the surface interaction of *E. coli* with dye. Improvement in PL intensity based on the multipoint interaction between the dye and the different external cell membrane structures present in Gram-negative bacteria (*E. coli*).

#### Preparation of artificial urine

**Figure 6:**
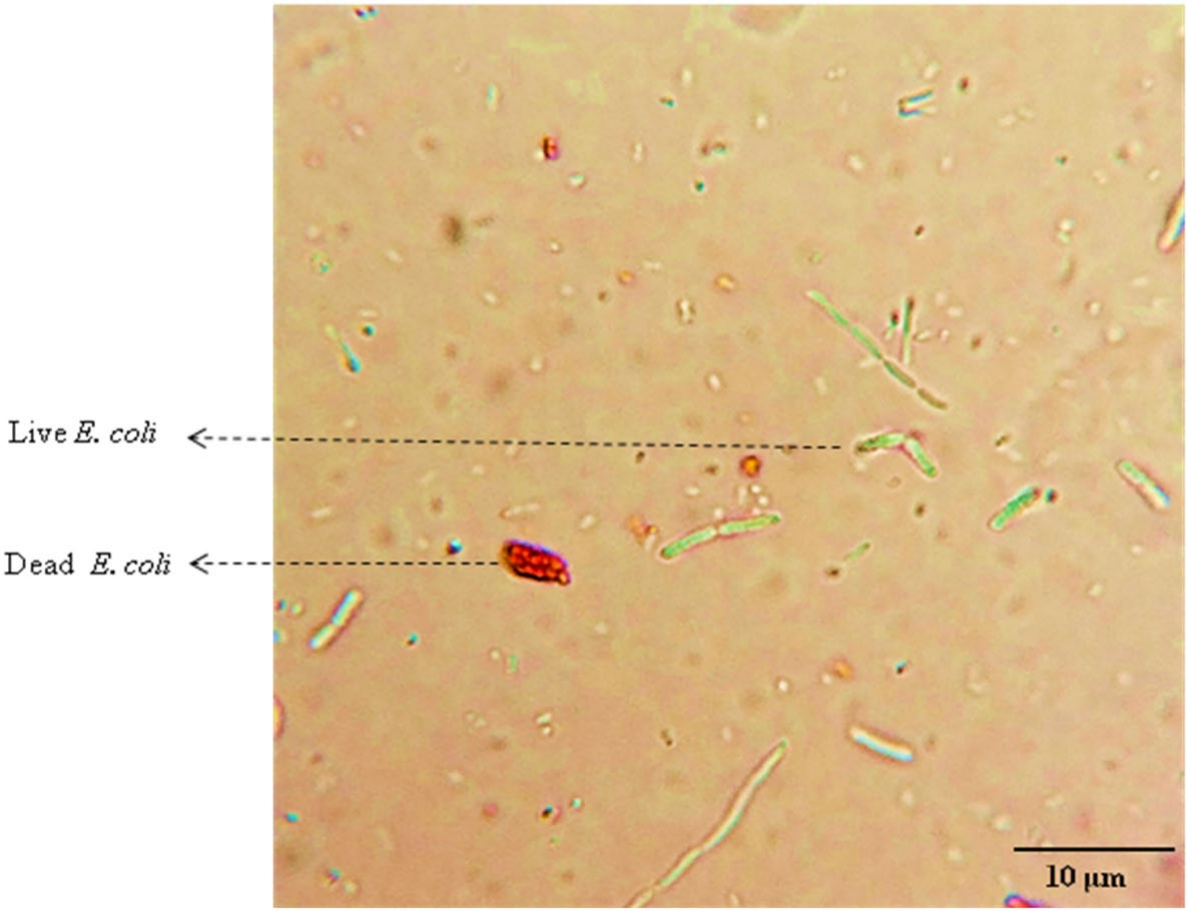
Microscopic image (×100) shows the fluorescence from live *E. coli cells* (under UV exposure) and stains from dead *E. coli* cells stained with 3 mg mL^−1^ MEH-PPV solution + 0.5% PVA.

In order to improve the interaction efficiency of MEH-PPV with bacteria, an artificial urine solution^39^ was prepared and 5 mL of MEH-PPV solution (3 mg/mL) and 0.5% of PVA was added. A red coloured solution was obtained. The mixed bacterial suspension comprising of *S. aureus* (Gram positive bacteria) and *E. coli* (Gram negative bacteria) with the equal count (10^4^–10^5^ CFU/mL) were added to the obtained solution. When the solution mixture was viewed through the microscope under the illumination of UV radiation, a green emission and a red coloured stain was observed (depicted in Fig. 6).

From the rod shape and size of the bacteria, it is confirmed that the green coloured emission is the fluorescence from *E. coli* which are live and absence of luminescence from dead *E. coli* cells. The viability of organisms was evidently observed with appropriate movements of the mixed cultures. A few cells of *S. aureus* were also captured with luminescence analogously less than *E. coli*.* T*he above study confirmed that the MEH-PPV solution can detect and differentiating between live and dead cells.

#### Suitability of MEH-PPV solution for cell imaging efficiency of various pathogens

To evaluate and compare the in vitro cell imaging efficiency of MEH-PPV, ATCC strains, namely *E. coli*, *S. aureus*, and *C. albicans*, were stained with MEH-PPV and different commercial dyes. Colonies from overnight culture plates are used for all the staining methods. Gram staining is an important and distinctive primary identification in microbiology laboratories. Interpretation of gram staining was completed with purple/violet staining for gram positive organisms and pink or red staining for gram negative organisms corresponding to cell wall structures. Rhodamine 6G (Rh 6G) is a highly fluorescent, lipophilic dye that is widely used as a fluorescent probe for measuring water flow and direction.

**Figure 7:**
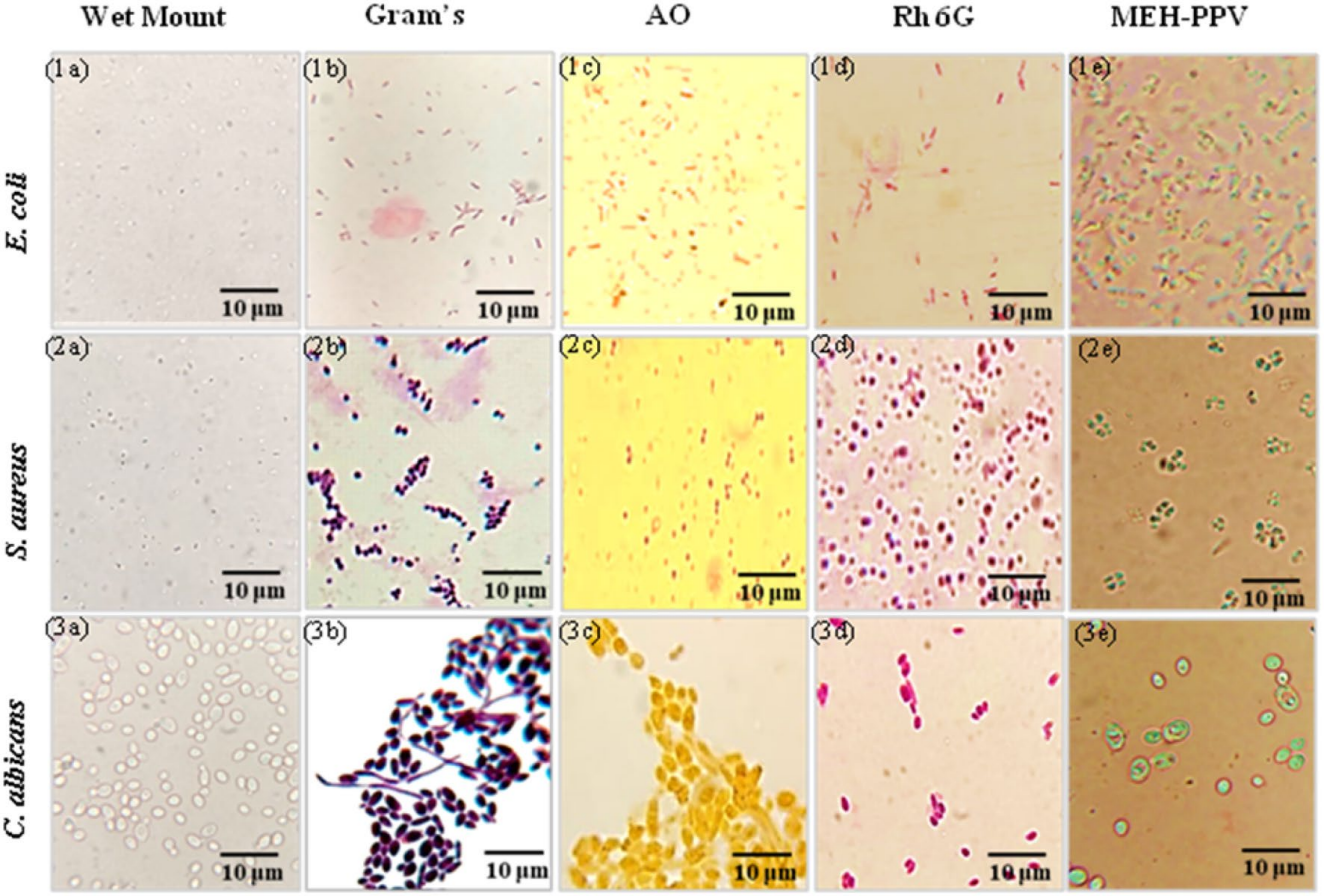
Light microscopic images of unstained (**1a**–**3a**), Gram’s stain (**1b**–**3b**), Acridine Orange (**1c**–**3c**), Rhodamine 6G (**1d**–**3d**), and MEH-PPV (**1e**–**3e**) stained *E. coli* (**1a**–**1e**), *S. aureus* (**2a**–**2e**), and *C. albicans* (**3a**–**3e**).

Light microscopy of pathogens stained with rhodamine 6G shows a bright red to pink colour. Acridine orange (AO) is a fluorescent dye that binds to pathogen nucleic acids. AO fluorescence microscopy usually shows a bright orange colour, but faint staining (pale orange) can be seen under light microscopy. *E. coli*, *S. aureus*, and *C. albicans* are stained with fluorescent (AO and Rh 6G) and non-fluorescent (Gram’s) stains, as shown in Fig. 7 (1a–3e).

A wet preparation (1a-3a) of each ATCC strain is also provided for staining differentiation of cells. Brightfield images of the prepared slides show the corresponding staining in terms of the indicated colours. Wet preparations show only clear cell morphology. MEH-PPV staining of *E. coli*, *S. aureus*, and *C. albicans* provides a different approach to organism-to-organism interactions. Here *E. coli*, *S. aureus*, and *C. albicans* showed bright and uniform luminescence in all cells with minute change in emission. The cell wall composition of these three group of pathogens plays a vital role in the emission pattern. *S. aureus* has a thick peptidoglycan layer in its cell wall. *C. albicans*, being yeast like fungal organism, doesn't have a peptidoglycan cell wall, but its cell wall contains other complex polysaccharides and glycoproteins. In addition to the thin peptidoglycan, the outer membrane of Gram-negative bacteria like *E. coli* contains lipopolysaccharides (LPS) on its surface. These components may retain the stains and appear with specific staining conditions. Thus the validity obtained shows that the efficiency of optimized MEH-PPV solution is more compatible with synthetic dyes and allows multimicrobial pathogen detection. The staining capability of MEH-PPV solution was also evaluated with mixture of known concentrations of *E. coli*, *S. aureus*, and *C. albicans* (Referred in the supplementary document—Fig. S1).

## Conclusion

In summary, we have synthesized MEH-PPV light emitting polymer via gilch route polymerization and characterized. Step wise procedures followed to study the microbial sensing nature of MEH-PPV. Started with the solvent selection, toluene was used as solvent to dissolve MEH-PPV since it keeps the bacterial interaction live upto maximum of time. Further the solution concentration of MEH-PPV was optimized for the selective staining of *E. coli*. Among three different concentrations tested, 3 mg/mL found to be more prominent for the selective staining of the bacteria. Once the visual observation proven that the material has the interactive capacity with the bacterial cell, this is further confirmed through the PL spectrum by combing the results of MEH-PPV solution with and without *E. coli* suspension. The results obtained shows two different peaks due to hydrophobic nature of the synthesized material. In order to improve the water solubility, the red solution mixture was prepared by adding the components for artificial urine medium, 0.5% of PVA, and solution of MEH-PPV. The resulted solution was used for the validation of mixed microbial population. Consequently, the cell imaging efficiency of synthesized MEH-PPV was tested along with some of the commercially available dyes and microscopic observations were captured. Over all, this proposed study showed that the conjugated polymers could potentially be used as an effective fluorescent dye for the selective identification of live *E. coli* cells. Structural modifications of MEH-PPV conjugated polymer have required making them soluble in water and thus will results in improved interaction with bacteria. Studies on quantification of bacterial cells based on intensity values would be of great extent to make it convenient for the sensitive detection.

This study represents the first investigation of MEHPPV as a fluorescent polymer for UTI pathogen detection and the findings presented here are the first-of-their-kind in the scientific literature. To the best of our knowledge, no prior research has explored the application of MEHPPV specifically for UTI pathogen detection. This work serves as a pioneering effort to harness the unique properties of MEHPPV in the context of UTI diagnostics, aiming to fill the existing gap in literature regarding the use of fluorescent polymers for UTI pathogen detection.

### Supplementary Information

Supplementary Information.

## Data Availability

The datasets used and/or analysed during the current study available from the corresponding author on reasonable request.
