# Supplementary material for: Fluorescent polymer as a biosensing tool for the diagnosis of microbial pathogens
Source: Sci Rep. 2024 Jan 25;14:2203. doi: 10.1038/s41598-024-51919-6 (PMC10810778; doi:10.1038/s41598-024-51919-6)
Supplement: Supplementary file 1 — Supplementary Information. [file 41598_2024_51919_MOESM1_ESM.docx]

**FLUORESCENT POLYMER AS A BIOSENSING TOOL FOR THE DIAGNOSIS OF MICROBIAL PATHOGENS**

Selvi Krishnan^a^, Stephen Jose^a^, K Periyasamy Bhuvana ^a*^, Angayarkanny S^b^, and Joseph Bensingh R^a^

*^a^Central Institute of Petrochemical Engineering and Technology, Chennai, India.*

*^b^Department of Chemistry, Anna University, Chennai, India.*

**Email:* [*kpusha27@gmail.com*](mailto:kpusha27@gmail.com)

**Figure S1:**


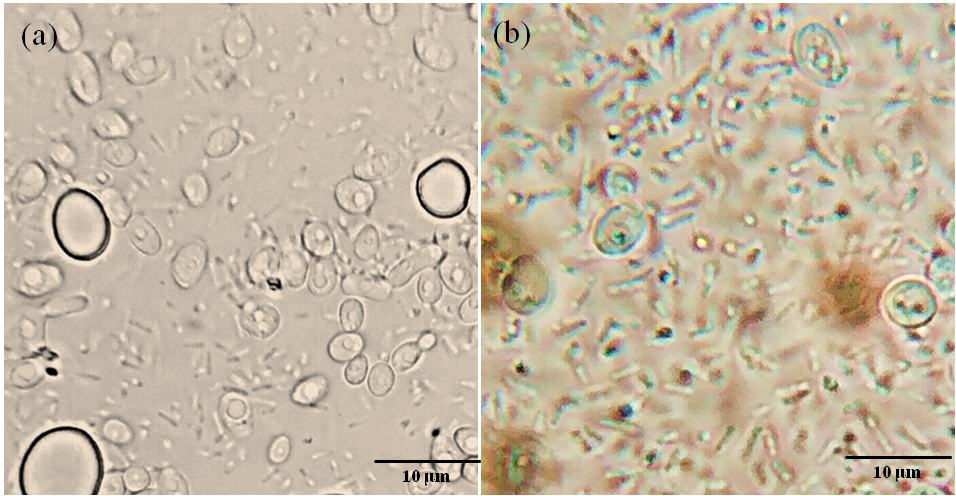


Fig S1: The microscopic examination (100x) of interaction of multimicrobial population of *E. coli, S. aureus, and C. albicans* without (a) and with (b) optimized MEH-PPV solution (3mg/mL).

**Figure S2:**

Fig S2: The microscopic examination (100x) of *E. coli* after incubating with 3mg/mL solution of MEH-PPV. Fig S2 (a) depicts the highest concentration of *E. coli* cells that is upto 10^5^ CFU/mL and Fig S2 (b) represents the lowest concentration of 10 CFU/mL.
